# Determinants of community-led ivermectin treatment adherence for onchocerciasis control in Western Ethiopia: a case-control study

**DOI:** 10.1186/s41182-020-00210-1

**Published:** 2020-04-20

**Authors:** Fikadu Ayalew, Desta Debalkie Atnafu, Melkamu Bedimo, Kebadnew Mulatu

**Affiliations:** 1 School of Public Health, College of Medicine and Health Science, Bahir Dar University, Bahir Dar, Ethiopia; 2 Department of Health System and Health Economics, School of Public Health, College of Medicine and Health Science, Bahir Dar University, Bahir Dar, Ethiopia; 3 Department of Epidemiology and Biostatistics, School of Public Health, College of Medicine and Health Science, Bahir Dar University, Bahir Dar, Ethiopia

**Keywords:** Onchocerciasis, Determinants, Adherence, Ivermectin, Ethiopia

## Abstract

**Background:**

Onchocerciasis is an infection of a filarial worm which is endemic in Sub-Saharan Africa, including Ethiopia. Annual mass treatment with high coverage over for a long period of time should lead to a complete interruption of transmission and the ultimate elimination of the parasite. However, in Ethiopia, the required coverage levels were not achieved. Thus, the aim of this study was to identify the possible determinants of onchocerciasis treatment adherence in Assossa District.

**Methods:**

A case-control study was conducted among 528 respondents (176 cases and 352 controls). Cases were respondents who took all five doses of treatments, and controls were those who took at most four does of ivermectin treatments (missed at least one or more doses). Structured questionnaire was used for data collection. Each possible factor for treatment adherence, with a *P* value < 0.2 obtained in the bi-variable logistic regression was entered into the multivariable logistic regression models to control the confounding factors. *p* value < 0.05 was used as cut-off-point for a variable to become a significant determinant of treatment adherence in multivariable logistic regression.

**Results:**

Participation in selecting drug distributers [AOR = 2.7, 95%CI (1.7–4.1)], measuring height for dose determination [AOR = 3.6, 95%CI (1.9–6.7)], perceived risk of getting onchocerciasis [AOR = 2.1, 95%CI (1.6–2.7)], living near running water [AOR = 1.7, 95%CI (1.1–2.8)], and perceived needs of support for intake of ivermectin [AOR = 3.2, 95%CI(2.1–4.9)] were independent predictors for t treatment adherence.

**Conclusions:**

Treatment adherence was influenced by participation in selecting drug distributers, measuring height for dose determination, perceived risk of getting onchocerciasis, living near running water and perceived needs of support for intake of ivermectin. To improve intake of the drug and its adherence, the community should be empowered to make decisions, and counseling family members and sensitizing those living far from river sides is commendable. Health information about onchocerciasis should be strengthening to increase risk perception.

## Introduction

Onchocerciasis commonly called river blindness (RB) is caused by a parasitic infection of filarial worm, *Onchocerca volvulus* [1]. The worm is spread by the repeated bites of black fly while feeding on blood meal [1–3]. The flies are mostly found in remote agricultural area around rapidly flowing rivers, a suitable habitat for black fly to breed [2, 4, 5].

Onchocerciasis is a public health problem in tropical climate regions. Globally, more than 123 million people are living in endemic areas and at risk of developing onchocerciasis infection. It was also reported that above 25 million people are contracting the disease [5]. Though the distribution of the disease occurrence varies widely across regions, more than 99% of people contracting the disease were found in 30 Sub-Saharan African countries, including Ethiopia [2, 4–6].

The World Health Organization (WHO) has currently recognized the disease and included it in the list of neglected tropical diseases. Onchocerciasis is the second infectious cause of blindness next to trachoma [2]. Around 1–2 million people were visually impaired because of RB infection [1, 2, 7]. It could also decrease life expectancy by 7–12 years of the most socio-economic weaken and debilitated adults [1, 8–10]. The rate of infection in highly endemic areas rose to 80–100 % for the age of 20 years. Ethiopia is among the endemic countries suitable to harbor two species of black fly [11]. In the country, more than 16.3 million people are at risk of RB and 5 million people are affected by the disease [11, 12].

The disease was highly prevalent and a major public health problem in endemic foci of Ethiopia and early prevention of the disease using immunization is impossible [4]. The most applicable mechanisms of prevention of RB infection was vector control measures (using personal protective wear), and administering preventive chemotherapy with ivermectin for vulnerable people [4]. According to the national onchocerciasis illumination strategy targeting 2020, only 184 are eligible and selected for bi-annual treatment with the drug out of 188 endemic districts [13]. The area where the study took place was also parts of the strategy and five rounds of treatments were provided [14]. In Ethiopia, since mass drug administration has been started in 2001, more than 19 rounds of treatments have been administered [15].

Several literatures showed that mass treatment of onchocerciasis with ivermectin resulted in reduction of transmission. The administration of ivermectin in endemic areas of Ethiopia led to a significant reduction of vector infectivity. However, a revival of the transmission has been noticed because of not adhering for ivermectin treatment as much time as the lifespan of adult *Oncocerca volvulus* worm (10 years and above) [16–18]. Until 2013, the coverage of treatment supplementation in the country was limited and it was addressed only 20% of endemic focci from target. In 2013, following support by partners, about 75 % of endemic districts in the country (12.3 million people) were covered for onchocerciasis treatment though this coverage was lower than WHO recommendations [3, 5]. Ethiopia has been implementing a strategy of eliminating onchocerciasis through biannual administration of ivermectin treatment [19].

Benshangul Gumuz Region, where the study had taken place, is an endemic to onchocerciasis. Though significant amount of treatment campaign was conducted in the region, about 42.3% of micro filaricide prevalence was reported. This is even the contribution of residual transmission that has resulted after treatment has already been administered. Additionally, this prevalence was also exacerbated because of the fact that the lower annual coverage of treatment (64.3%) and the presence of non-adherents in the treatment campaign (43%) [20, 21]. This coverage and adherence rate was vastly deviate from that of the national plan and the WHO target for onchocerciasis control [5].

Various theories and studies suggested that adherence to onchocerciasis treatment with ivermectin was associated with sex [21, 22], ethnicity [21, 23], age [21–23], length of stay [21, 23], familiarity of community-directed drug distributer [23], fear of drug side effects [21], perceived needs of support for intake of ivermectin [22, 23], perceived performance of community drug distributer [21, 23, 24], religion [22], education [22], risk perception to oncocerciasis [23], measuring height the best way to determine a person’s treatment dose [24], educational level [25], and perceived effectiveness of onchocerciasis treatment with ivermectin [21, 23].

Onchocerciasis control needs 90% treatment coverage of ivermectin [9]. Though CLTI adherence plays a key role in the success of onchocerciasis control, sustained attention is not given in endemic area of Ethiopia. Unless adherence to the drug administration is guaranteed, the control of onchocerciasis would not be effective. Benshangul Gumuz is one of the onchocerciasis endemic foci in Ethiopia where ivermectin supplementation was initiated. In the region, residual transmission has been noticed because of poor treatment adherence. Failure of ivermectin treatment adherence is the bottle neck to control the disease. However, factors influencing CLTI adherence in the study area was not yet well studied. Thus, this study aimed to identify determinants of adherence of CLTI for onchocerciasis control among individuals in Assossa District, Western Ethiopia.

## Methods

### Study design, area, and period

A community-based un-matched case-control study was conducted in Assosa District, Benshangul Gumuz, Western Ethiopia. The study was implemented between April and May 2017. Assosa is the capital of Benshangul Gumuze Region and is located in the west of Ethiopia, 675 km far from Addis Ababa. The area is found with an altitude between 560 and 1570 meters above sea level. The average annual temperature is 27 °C. Around 75% of the district is hot spot area and suitable to harbor the worm. The estimated total populations for the year 2016/2017 were 108,693 [26], as projected from 2007 population and housing census. As a strategy of onchocerciasis control, an organized annual mass drug administration has been in progress in the district since 2013 by APOC, Ministry of Health and in connection with the district health office administration. Until 2017, about five treatment rounds have been already administered and records were maintained on yearly treatment registration books. Accordingly, around 46,738 people had been benefited to the treatments administered in the five rounds. Rivers found in the district include Yabus, its tributaries, the Buldidine, Afa, and Amba-Anid. The main economic activities of the district are agriculture, cattle breeding, and traditional gold panning/digging, as well as trading [14].

### Study participants

All the individuals who lived in onchocerciasis endemic villages of Assosa District were included in the study. However, pregnant mother, under 15 children, those who permanently left the area and new comers to the district after the first treatment round had completed as well as sick persons were not considered. Cases were those adherents, whose name or identification number had been listed on the APOC’s treatment register books and had taken all the five yearly doses of ivermectin. Whereas controls were, non-adherents, whose name or identification number had been registered in the APOC’s treatment register books during the first treatment round (2013), and missed at least one doses of treatment (2017). People found in the study locality and recorded on the APOC’s registering log book of the 2013 (first treatment round) were considered as sampling frame or source population.

### Sample size determination, sampling technique, and procedures

The sample size was calculated using the double population proportion formula using Epi-Info version 7 software on the basis of the following assumptions: 95% confidence interval (CI), 80% power, 1:2 ratio of cases-to-controls, odds ratio of 2 [16], and design effect of 2. Thus, by considering the proportion of perceived measuring height to dose determination 87.4% for cases and 97.8% for controls, the final sample size was determined to be 178 for cases and 356 for controls (a total of 534 respondents) after adding non-response rate of 10%. The sample size was calculated for exposure status of different variables: perceived measuring height to dose determination, perceived risk of contracting onchocerciasis, knowing mode of transmission, and educational status. We selected the largest sample among these exposure variables. Simple random sampling technique was employed to recruit study respondents. Fifteen out of 74 villages were selected using lottery methods. The sample size calculated was allocated to these villages using probability proportional to size, where numbers of individuals registered in the APOC’s treatment register book were used as a measure of size. The required numbers of respondents in each of the selected villages were drawn by tables of random numbers generated by computer.

### Data collection tools, procedures, and quality assurance

The data were collected using structured questionnaires through face-to-face interviews. The questionnaire was prepared in English, then translated into Amharic and back translated to English to check its consistency, and pretest was done prior to data collection. The data were collected through face to face interviewer administered questionnaire. Adherence to ivermectin is the degree in which eligible individuals in the endemic villages to onchocerciasis get ivermectin treatment in each of the annual treatment campaign held since 2013, the introduction of the program in the district up to 2017. An entity was labeled as adherent if he or she was registered on APOC’s registration book since 2013 (the first treatment round), and got all five doses of ivermectin. Whereas, an individual who had registration book since the first treatment round and missed at least one of the five doses of ivermectin was considers as non-adherent. Knowledge about onchocerciasis and perceived risk of getting onchocerciasis were measured using 13-point knowledge score and 6 point perception score questions respectively. Each correct response was given a score of 1 for right responses and wrong responses a score of 0. The questionnaires included risk factors, prevention, susceptibility, adherence, benefit of ivermectin treatment, etc. Respondents with total score greater than or equal to the mean value were categorized as “good” and those who score less than the mean were categorized as “poor.” Data were collected by diploma holder experts of health field supervised by professional nurse. To assure the quality of data, three days training, pretest and frequent supervision with on spot checking of questionnaires was conducted.

### Data processing and analysis

Data were checked, coded, and then entered into Epi-Info software and export to SPSS for statistical analysis. Simple descriptive statistics were used to summarize the profiles of the study respondents. Binary logistic regression model was applied to identify significant variables. First bi-variable binary logistic regression model was fitted, then candidate variables with *p* value < 0.2 were entered into multivariable binary logistic regression models to identify factors significantly associated with ivermectin treatment adherence. Hosmer and Lemeshow goodness of fit test with *p* value > 0.05 was computed to test the model fitness. Adjusted odds ratio (AOR) with 95% CI and *p-* value < 0.05 was used to identify significant independent determinants of ivermectin treatment adherence.

### Ethical considerations

The study was conducted following permission letter had obtained from ethical review committee of Bahir Dar University (reference number: APHI/01/742). Support letter was taken from the Benshangule Gumuz Regional Health Bureau, Zonal and District health offices accordingly. Both verbal and written consent were taken from each study respondents. The personal profile of respondents was not written on the questionnaire and all information obtained from the respondents was kept confidential.

## Results

### Background characteristics

A total of 534 study subjects were included, resulting over all response rates of 98.9% (98.9% both for cases and controls). The mean age of the respondents was 37.3 years (SD ± 13.8 years). About 69.1% of the respondents were Muslims. More than half (59.3%) of the respondents were farmers by occupation. Over all 51.9% of the respondents (43.8% for cases and 55.9% for controls) were females. The mean age of cases was 35.8 years, while it was 32.0 years in controls. A higher proportion of Amhara ethnic group (69.9%, 76.9%), Muslims (70.5%, 68.5%), farmers (62.5%, 57.7%), and married (82.4%, 71%) were observed in both groups of cases and controls respectively. However, there is no statistical difference in the likelihood of such variables in the interest of treatment adherence (Table 1).

**Table 1:** Background characteristics of respondents and bi-variable analysis in Assosa district, Western Ethiopia, 2017.

Variables	Exposure status	Cases	Controls	COR	95% CI
Age	15—34	83 (47.2)	213(60.5)	0.6	0.4–0.84
	≥35	93 (52.8)	139 (39.5)	1	
Sex	Female	77(43.8)	197(55.9)	0.6	0.43–0.9
	Male	99(56.2)	155(44.1)	1	
Ethnicity	Oromo	12(6.8)	25(7.1)	0.19	0.05–0.7
	Amhara	123(69.9)	269(76.4)	0.18	0.06–0.6
	Berta	31(17.6)	54(15.4)	0.22	0.07–0.8
	Tigray	10(5.7)	4(1.1)	1	
Educational level	Unable to read and write	91(51.7)	156(44.3)	1.33	0.8–2.14
	Elementary	50(28.4)	116(33)	0.983	0.6–1.7
	Secondary and above	35(19.9)	80(22.7)	1	
Participation during selection of CDD	Yes	126(71.6)	197(56)	1.983	1.3–2.9
	No	50(28.4)	155(44)	1	
Measuring height for dose determination of Ivermectin	Yes	164(93.2)	289(82.1)	2.979	1.6–5.7
	No	12(6.8)	63(17.9)	1	
Support by family and CDD during intake of Ivermectin	Yes	118(67)	158(44.9)	2.498	1.7–3.7
	No	58(33)	194(55.1)	1	
Perceived risk of getting onchocerciasis	Yes	96(54.5)	159 (45.2)	1.457	1.01–2.1
	No	80(45.5)	193 (54.8)	1	
Living near running river water leads risk of getting onchocerciasis	Yes	146(83)	218 (62)	2.99	1.9–4.68
	No	30(17)	124 (38)	1	
Knowing mode of transmission	Yes	170(96.6)	277(78.7)	7.671	3.3–18
	No	6(3.4)	75(21.3)	1	
Knowing onchocercias is preventable	Yes	170(96.4)	212(88.6)	3.63	1.5–9.8
	No	6(3.4)	40(11.4)	1	

### Factors associated with ivermectin treatment adherence

The bi-variable logistic regression showed that only educational status (*p* = 0.14) was not significantly associated with adherence of ivermectin (Table 1).

To identify the independent determinants for adherence of ivermectin, a multivariable logistic regression model was fitted. After adjustment for possible confounders, participating during the selection of community drug distributers (CDDs) [AOR = 2.7, 95% CI (1.7–4.1], measuring height for dose determination of ivermectin [AOR = 3.6, 95% CI (1.8–6.6)], supports from family and CDDs during intake of ivermectin [AOR = 3.2, 95% CI (2.1–4.9)], perceiving risk of getting onchocerciasis [AOR = 2.1, 95% CI (1.6–2.7)], living near running river water [AOR = 1.7, 95% CI (1.1–2.8)], knowing mode of transmission [AOR = 6.3, 95% CI (2.7–15)], and knowing onchocerciasis is preventable [AOR = 2.7, 95% CI (1.1–6.6)] were the most important factors that increased adherence of RB treatment with ivermectin. However, age, sex, ethnicity, and educational status did not appear in the final model (Table 2).

**Table 2:** Determinants of ivermectin adherence among beneficiaries in Assosa District, Western Ethiopia, 2017

Variables	Exposure status	Cases	Controls	COR	(95% CI)	AOR	(95% CI)	*p-* value
Participation during selection of CDD	Yes	126(71.6)	197(56)	1.983	1.3–2.9	2.66	(1.7–4.1)	0.0001*
	No	50(28.4)	155(44)	1		1		
Measuring height for dose determination of ivermectin	Yes	164(93.2)	289(82.1)	2.979	1.6–5.7	3.6	(1.7–6.6)	0.001*
	No	12(6.8)	63(17.9)	1		1		
Support by family and CDD during intake of ivermectin	Yes	118(67)	158(44.9)	2.498	1.7–3.7	3.21	(2.1–4.9)	0.0001*
	No	58(33)	194(55.1)	1		1		
Perceived risk of getting onchocerciasis	Yes	96(54.5)	159 (45.2)	1.457	1.01–2.1	2.1	(1.6–2.7)	0.001*
	No	80(45.5)	193 (54.8)	1		1		
Living near running river water leads risk of getting onchocerciasis	Yes	146(83)	218 (62)	2.99	1.9–4.68	1.7	(1.1–2.8)	0.0001*
	No	30(17)	124 (38)	1		1		
Knowing mode of transmission	Yes	170(96.6)	277(78.7)	7.671	3.3–18	6.3	(2.7–15.)	0.001*
	No	6(3.4)	75(21.3)	1		1		
Knowing onchocercias is preventable	Yes	170(96.4)	212(88.6)	3.63	1.5–9.8	2.68	(1.2–6.6)	0.032*
	No	6(3.4)	40(11.4)	1		1		

*CDD* = community drug distributor

*Significant variables at *p* < 0.05

## Discussion

Mass drug administration (MDA) of microfilaricidal drugs such as ivermectin is the backbone of efforts made to eliminate onchocerciasis. However, long-term and large-scale supplementation of the drug itself (alone) has failed to address elimination. It is important to guarantee high levels of adherence for ivermectin uptake so as to obtain elimination targets. Thus, the need to identify factors contributing to the adherence for CLTI is paramount for efforts exerted in the prevention and control of onchocerciasis which is the commonest economic and public health problem in the endemic foci.

Community involvement, ownership, and favorable attitude of the CLTI are related with an improvement of the MDA coverage and adherence which halts transmission and reduces the burden of onchocerciasis [27–30]. The respondents who involved in the selection of CDDs were more likely to adhere to the ivermectin treatment as compared to those who did not participated in the selection [16]. This could reflect the recommendations by APOC: in the selection of CDDs, and in the choice of the period in which ivermectin distribution has to be undertaken; the community members should be involved so as to insure community empowerment, participation, ownership and sustainability of the program [31]. Other literatures also found that community perception on the CDDs’ knowledge, role, medical training, and performance in CLTI for onchocerciasis control empirically influences adherence to ivermectin mass treatment [7]. This could be attributed to the fact that individuals who were not invited to participate in the selection of CDDs believed that they are less considered and empowered in decision-making during implementation of the program provided that poor treatment adherence. This was also the fact that in the absence of genuine participation, the communities could perceive that inefficient and less knowledgeable CDDs might be selected and resulted in undesirable treatment administration which believed not healthful.

 Previous studies revealed that inhabitants in the endemic corridors of RB whose age 5 years and above are advisable to obtain doses of ivermectin proportional to their measurements of body height [4, 7, 16, 32]. In line with the above fact, we found that perceived measuring height as best mechanism of estimating amount of dosage of ivermectin for administration during treatment campaign was a determinant for ivermectin treatment adherence. On the other hand, non-adherent subjects in the studied community feared risk of being overdosed and toxicity if height instead of weight is used as a proper way of calculating doses for ivermectin. This could also be explained that extensive labor exhausted in the farm by the community at the time of campaign might cause physical weakness and loss of body weight provided that the same height condition and for this reason determining level of doses based on height measurements was wrongly perceived of being intoxicated. The conviction is also linked to the trend shown from dosage determination in the case of malaria treatment which is estimated with respect to weight instead of height and resulted in confusion for non-adherents. Therefore, it is suggested that continuous sensitization of CDDs and education of illegible community, how dosage could be determined for ivermectin mass treatment administration. Furthermore, expanding health education can augment information about the onchocerciasis and the demand for prevention and control. This was vividly presented in the results of current study in that knowing modes of transmission of infection due to the bites of black fly was significantly associated with ivermectin adherence. Thus, the program should be prioritized and contained in the national health education, communication, and transformation priorities applicable for endemic localities.

Encouragement by family and community drug distributors during intake of ivermectin was significantly associated with annual ivermectin treatment adherence. This is consistent with other study found in Southwestern Uganda [5, 32]. A study conducted elsewhere showed that parent involvement in the CLTI process was one of the factor that increased the adherence [33, 34]. Other findings also revealed that the majority of respondents who adhered were encouraged for ivermectin intake by their family and CDD [25, 26, 35]. Provided that family support is the basic element of social system and coalition, it allows information dissemination and explanations in relation to the benefit, contra indication, drug availability, and safe administration modalities of the anti-microfilariae drug. Such kind of communication could empower individuals to make healthy decision.

The odd of ivermectin treatment adherence was 2.1 times more in individuals’ perceived threat of contracting onchocerciasis as serous disease. This finding is compliment with other scholarly findings [16, 22, 23, 25, 28, 32, 33, 36]. This might be attributed to the fact that if communities’ vulnerability to onchocerciasis is anticipated to be high then the uptake of the CLTI program is also high. When people believe that personal susceptibility to onchocerciasis is high then adherence to the treatment as well is high. Another study also strengthen this finding, most respondents (90.9%) believed that onchocerciasis was a serious disease and for these reason about 52.8% of respondents were adhered the program [26].

In endemic localities of RB, communities’ hesitation to inhabit and dwell around fertile riverside (bank) is linked with black fly infestation [37–39]. Likewise, in this study, living around river basin was an independent determinant for CLTI adherence. A kind of community living condition therefor, increased utilization of annual treatments with ivermectin, as likely supported by findings conducted elsewhere [4, 32, 39]. Fast flowing river water bed is the commonest suitable ecological zone in which flies are harbored and breed. Black fly is a known vector, which carries an ethioletic agent of onchocerciasis through infected blood while fed on blood.

In Africa and endemic localities, community-led mass administration of ivermectin has become the primary and known intervention for onchocerciasis eradication next to control of the vector via regular entomological surveillance [25, 40]. This is similarly reported by the current study in that ivermectin is the best preventive modality of onchocerciasis.

The odds of mass ivermectin treatment adherence in individuals who knew the mode of transmission of onchocerciasis was 6.3 times higher than those did not know. This result was in line with scholarly findings in other locality [24]. This might be explained in that individuals who knew about the transmission cycle of the disease want to be safe by considering themselves in the treatment regimen of ivermectin given the existing side effects and contraindications of the drug.

This study could not be realized without any limitations. Among them recall bias was the commonest one. Probing technique was applied and incident cases were included during data collection in order to decrease the effect of such bias. The non-computerized registration system of APOC’s applied might have poor estimate of cases and controls for the study. The qualitative study was not employed and therefore, future researchers is recommended to apply mixed methods since qualitative data would have been more appropriate to understand the perspectives of the non-adherers in such research context. The results of the study may only generalizable to those localities with similar ecological conditions.

## Conclusion

Control of onchocerciasis from endemic foci in Ethiopia was influenced by different factors that have relation with ivermectin treatment adherence. Community engagement in selecting CDDs, using height for dose determination of ivermectin, supports from family and CDDs, perceived risk of getting onchocerciasis, living near running river water, knowing mode of transmission, and knowing onchocerciasis is preventable were the major factors for increased adherence of ivermectin treatment. To improve intake of the drug and its adherence; the community should be empowered to make decisions, and counseling of family members and sensitizing those living far from river sides is commendable. Health education on onchocerciasis, its mode of transmission, and its treatment should be strengthening to increase risk perception. The CLTI program should involve and empower community members to make major decisions and direct the distribution of ivermectin for a sustained period of years. Among the decisions should be managed by the community includes seasons of drug distribution; mode of distribution (whither village-to-village or at center of village); people who manage distribution and selection of the community implementers also known as community-directed distributors (CDDs). In general, with the current level of adherence practice, the likelihood for the program to achieve elimination of the disease by 2020 targeted by the country and WHO is quite impossible unless the significant factors were given due emphasis in policy and program framework.

## Data Availability

Data of this study are available without restriction. Contact to this e-mail: destad2a@gmail.com when needed.

## Abbreviations

CDDs: Community drug distributers
COR: Crude odds ratio
AOD: Adjusted odds ratio
CI: Confidence interval
CLTI: Community-led treatment with ivermectin
MDA: Mass drug administration
RB: River blindness
APOC: African Program on Onchocerciasis Control
WHO: World Health Organization
SD: Standard deviation
^o^C: Degree centigrade
CDC: Centers for Disease Control and Prevention
PHEM: Public health emergency management
EPHI: Ethiopian Public Health Institute
